# Identification and expression analysis of maize *NF-YA* subunit genes

**DOI:** 10.7717/peerj.14306

**Published:** 2022-11-07

**Authors:** Mingyue Lv, Hongzhe Cao, Xue Wang, Kang Zhang, Helong Si, Jinping Zang, Jihong Xing, Jingao Dong

**Affiliations:** 1 State Key Laboratory of North China Crop Improvement and Regulation, Hebei Agricultrual University, Baoding, Hebei, China; 2 Hebei Key Laboratory of Plant Physiology and Molecular Pathology, Hebei Agricultural University, Baoding, Hebei, China

**Keywords:** Maize, *NF-YA* subunit gene, Biological stress, Abiotic stress, Expression analysis

## Abstract

*NF-YAs* encode subunits of the nuclear factor-Y (NF-Y) gene family. *NF-YA*s represent a kind of conservative transcription factor in plants and are involved in plant growth and development, as well as resistance to biotic and abiotic stress. In this study, 16 maize (*Zea mays*) *NF-YA* subunit genes were identified using bioinformatics methods, and they were divided into three categories by a phylogenetic analysis. A conserved domain analysis showed that most contained a CCAAT-binding transcription factor (CBFB) _NF-YA domain. Maize *NF-YA* subunit genes showed very obvious tissue expression characteristics. The expression level of the *NF-YA* subunit genes significantly changed under different abiotic stresses, including *Fusarium graminearum* infection and salicylic acid (SA) or jasmonic acid (JA) treatments. After inoculation with *Setosphaeria turcica* and *Cochliobolus heterostrophus*, the lesion areas of *nfya01* and *nfya06* were significantly larger than that of B73, indicating that *ZmNFYA01* and *ZmNFYA06* positively regulated maize disease resistance. *ZmNFYA01* and *ZmNFYA06* may regulated maize disease resistance by affecting the transcription levels of *ZmPR*s. Thus, *NF-YA* subunit genes played important roles in promoting maize growth and development and resistance to stress. The results laid a foundation for clarifying the functions and regulatory mechanisms of *NF-YA* subunit genes in maize.

## Introduction

The nuclear factor-Y (NF-Y) transcription factor exists widely in eukaryotes and is also known as hemo-activator protein (HAP) (Thirumurugan et al., 2008; Petroni et al., 2012). NF-Y can bind to CCAAT-boxes in promoter sequences; consequently, it is also called CCAAT-binding factor (CBF) (Laloum et al., 2013). *NF-Y* is a large gene family composed of *NF-YA* (CBFB/HAP2), *NF-YB* (CBFA/HAP3) and *NF-YC* (CBFC/HAP5) subunits (Nardini et al., 2013). NF-Y is usually located in the nucleus and is evolutionarily conserved (Mantovani, 1999). In animals, NF-YA, NF-YB and NF-YC subunits are encoded by three single genes, and the three subunits function in the form of heterologous trimers (Benatti et al., 2008). In plants, the three subunits are encoded by more than 10 genes, and they can perform their functions independently (Petroni et al., 2012). The CBFB_NF-YA domain is the core conserved domain of the NF-YA family. The N-terminal of this domain can bind to NF-YB and NF-YC subunits, and the C-terminal can bind to DNA CCAAT-boxes (Quach et al., 2015). Compared with the rapid progress in mammalian and yeast (*Saccharomyces cerevisiae*) NF-Y protein-related research, research progress on the plant NF-Y family has been slow (Liang et al., 2012). Until now, this research has been limited to the preliminary bioinformatics comparisons between *A. thaliana* and related plant species, as well as the gene expression and function analyses.

The single subunit of *NF-Y* is widely involved in plant growth and development, such as controlling gametogenesis, embryo and plant development (Mu et al., 2013), abscisic acid (ABA) signal transduction (Yu et al., 2021), flowering cycle regulation (Hwang et al., 2019), primary root elongation (Ballif et al., 2011; Zhou et al., 2020), blue light response (Warpeha et al., 2007) and photosynthesis (Tokutsu et al., 2019) as well as stress responses, including to abiotic stresses such as drought, high temperature and salt (Li et al., 2021). The *NF-YA* subunit genes are involved in multiple processes in the plant lifecycle. In *Arabidopsis thaliana*, *NF-YA3* and *NF-YA8* genes mediate cell differentiation and embryo formation through the ABA signaling pathway during early embryonic development (Mu et al., 2013). *NF-YA1*, *NF-YA5*, *NF-YA6* and *NF-YA9* are involved in the development of gametes, embryos and seeds (Mu et al., 2013). *AtNF-YA5* is regulated by miR169, thereby improving the resistance of *Arabidopsis* to drought stress (Li et al., 2008). The overexpression of *OsNF-YA7* improves the drought tolerance of rice through an ABA-independent pathway (Lee et al., 2015). In potatos (*Solanum tuberosum* cv. ‘Desiree’), *NF-YA*s responds to drought by regulating the number of chlorophylls, stomatal conductance and photosynthesis (Li et al., 2021). *PtNF-YA9* plays an important role in the drought resistance of *Populus trichocarpa* as a positive regulator of stress resistance (Lian et al., 2018). There are five *NF-Y* genes in tomato (*Solanum lycopersicum*) that play roles in tomato fruit ripening (Li et al., 2016). In soybean (*Glycine max*), GmNFYC14 forms heterotrimer with GmNF-YA16 and GmNFYB2, activates *GmPYR1* mediated ABA signaling pathway and regulates soybean stress tolerance (Yu et al., 2021). In maize (*Zea mays*), ZmNFYB16 can form a heterotrimer with ZmNFYC17 and ZmNFYA01, and the heterotrimer binds to CCAAT cis-acting elements in the promoter region of stress response and growth-related genes through the *ZmNFYA01* subunit, regulating the expression of multiple genes related to stress resistance and growth, thereby improving the drought resistance of plants (Wang et al., 2018). ZmNFYA03 promotes early flowering by binding to the *FT-like12* promoter in maize (Su et al., 2018).

Plants have developed complex mechanisms to protect themselves against pathogens. Pathogenesis-related (PR) genes are the key elements of these mechanisms, and activated in response to pathogen attacks. They regulate production of several proteins, peptides or compounds which are toxic to pathogens or prevent pathogen infections where they start (Xie et al., 2010). The PR factors are thermostable, protease-resistant proteins of ~5–43 kDa which are expressed in all plant organs (Zribi, Ghorbel & Brini, 2021).

To date, an overall study of the maize *NF-YA* subunit gene family has not been reported, and the number, physicochemical properties and functions of maize *NF-YA* subunit genes are not clear. In this study, the maize *NF-YA* subunit genes were identified using bioinformatics methods, and their phylogenetic relationship, conserved domains, tissue specificity and gene expression patterns under biotic and abiotic stresses, such as salicylic acid (SA) and jasmonic acid (JA) treatments as well as disease resistance, were clarified. This would provide the foundation for elucidating the functions and regulatory mechanisms of maize *NF-YA* subunit genes.

## Materials and Methods

### Data sources and bioinformatics of *NF-YA* subunit genes in different species

The information and amino acid sequences of *NF-YA* subunit genes in maize, rice (*Oryza sativa*) and *Arabidopsis* were downloaded from MaizeGDB (http://www.maizegdb.org/), RGAP (http://rice.plantbiology.msu.edu/) and TAIR (https://www.arabidopsis.org/), respectively. The amino acid sequences were aligned using Clustal X software (Larkin et al., 2007). The aligned results were imported into MEGA 7.0 software (Kumar, Stecher & Tamura, 2016), and a phylogenetic tree was constructed using the maximum-likelihood method.

The chromosome location and the annotated information regarding the gene structure (including gene length, 5′-UTR, 3′-UTR and the distribution of each intron and exon) of maize *NF-YA* subunit genes were obtained from MaizeGDB, and their chromosome mapping was performed using RIdeogram software (https://cran.r-project.org/web/packages/RIdeogram/index.html). The gene structure map was drawn using IBS software (Liu et al., 2015).

The conserved domains of maize NF-YAs were analyzed using online software SMART (http://smart.embl-heidelberg.de/) and Pfam (http://pfam.xfam.org/), and the domain analysis maps were constructed using IBS software.

In accordance with the amino acid sequences of NF-YAs in maize, the subcellular localization was analyzed and predicted using Plant-mPLoc software (Chou & Shen, 2010).

For the predicted protein-protein interaction (PPI) analysis, all of the NF-YA amino acid sequences were searched using the STRING database version 11.5 (https://cn.string-db.org/) (Szklarczyk et al., 2021). A “confidence score” of STRING > 0.7 (high confidence) between proteins was used (Mei et al., 2021). The interaction networks of proteins generated using STRING were constructed to determine the relationships of proteins with NF-YAs.

### Existing data-based expression patterns of *NF-YA* subunit genes in maize

Using the SRA database in NCBI (https://www.ncbi.nlm.nih.gov/), the RNA-seq data of 31 maize tissues (such as Seed_5_days_after_pollination, Endosperm_25_days_after_pollination, Seed_10_days_after_pollination, *etc*.) and those obtained under both abiotic stresses, such as high temperature (14 days of maize seedlings cultured at 50 °C for 4 h), low temperature (14 days of maize seedlings cultured at 5 °C for 16 h), salt (14 days of maize seedlings irrigated with 300 mM NaCl for 20 h), ultraviolet (14 days of maize seedlings irradiated by ultraviolet lamp for 2 h), drought (14 days of maize seedlings dried filter paper covered for 4 h) and *Fusarium graminearum* infection (200 μL of *F. graminearum* conidial suspension was inoculated onto the internodes of maize at ten-leaf stage after punctured with a pipette), were downloaded. Using Hisat2 software (http://daehwankimlab.github.io/hisat2/), RNA-seq data was aligned to the reference genome of maize. Using Cufflinks software (http://cole-trapnell-lab.github.io/cufflinks/), the gene expression value expressed as Fragments Per Kilobase of exon per Million fragments mapped reads (FPKM) was calculated using standardized parameters of gene length and number of reads. The expression heat map of the *NF-YA* family genes in maize was constructed using HemI software (Deng et al., 2014).

### Plant materials and pathogenic fungal strains

The preserved seeds of maize inbred line B73, *Setosphaeria turcica* and *Cochliobolus heterostrophus* were from the Mycotoxin and Molecular Plant Pathology Laboratory, Hebei Agricultural University. *F. graminearum* strain PH-1 was provided by Prof. Mingguo Zhou at Nanjing Agricultural University. The *ZmNFYA01* Mu insertion mutant *nfya01* (Chr1, Insertion site 16041887, V4.0) and the *ZmNFYA06* Mu insertion mutant *nfya06* (Chr1, Insertion site 268308575, V4.0) were obtained from the ChinaMu Project (http://chinamu.jaas.ac.cn/) (Liang et al., 2019) and *nfya01* and *nfya06* plants used in this study were confirmed by PCR and quantitative real-time PCR (qRT-PCR) (Figs. S1 and S2). The primers for PCR and qRT-PCR identification are listed in Tables S1 and S2.

All the maize seeds were soaked in sterile water for approximately 24 h and potted in the mixture of vermiculite and nutrient soil at 1:1. The plants were cultured and grown in an artificial climate chamber with light for 14 h and darkness for 10 h at a temperature of 25–28 °C, and a humidity of 50–60%. The plants were irrigated once every 3 to 5 days, and the roots were irrigated with nutrient solution to ensure sufficient nutrition for the plants after reached the three-leaf stage.

*S. turcica* and *C. heterostrophus* were inoculated on Potato Dextrose Agar (PDA, 200 g/L potatos, 20 g/L glucose, 12 g/L agar) plates and grown in a 25 °C incubator.

*F. graminearum* was grown on PDA plates at 28 °C for 5 d, transferred to the carboxymethylcellulose sodium medium (CMC, 1.5 g/L CMC-Na, 1 g/L KH_2_PO_4_, 1 g/L NH_4_NO_3_, 1 g/L yeast powder and 0.5 g/L MgSO_4_·7H_2_O) and cultured in a shaker at 25 °C and 200 rpm for 5–7 d. The number of spores in the conidial suspension was counted using a blood cell counting plate, and more than 1 × 10^6^ spores were inoculated onto maize.

### Hormone treatments

In total, 1 L of salicylic acid (SA; 100 μM) and the same volume of jasmonic acid (JA; 100 μM) were sprayed independently and evenly on the aboveground parts of the two groups of maize B73 (five-leaf stage). Leaves were sampled at 0, 3, 9 and 24 h, frozen with liquid nitrogen and stored in a −80 °C refrigerator.

### Leaf inoculation of maize

The 4th and 5th leaves of seven-leaf stage maize plants were cut off, and a wound of 1.2 cm in diameter was cut every 8 cm using a disposable syringe needle to facilitate the invasion of pathogenic fungi. Tween 20 was applied to the wound, and cultured *S. turcica* and *C. heterostrophus* were punched into the wound to cover the leaf wounds. Two layers of fully wetted filter paper were placed at the bottom of a white culture box. The leaves inoculated with pathogenic fungi were placed into the box, and covered with preservative film. The box had several vents in the box and was maintained in the darkness at 25 °C. The filter paper was kept moist, and the changes in the lesions were observed every day.

A total of 4 or 5 days after inoculation, the inoculated leaves were stained with trypan blue. The prepared 0.5% trypan blue staining solution and the leaves of an appropriate size were placed in a 50-mL centrifuge tube and boiled for 15 min to stain the necrotic cells. Then, 100 g chloral hydrate was dissolved into 40 mL water to make the chloral hydrate decolorizing solution. The stained leaves were washed with water to remove the dye solution of trypan blue, and then placed into chloral hydrate solution and shaken for 2 to 3 days to discoloring fully. Image J software (https://imagej.nih.gov/ij/) was used to measure the lesion area caused by fungal infection. The experiment was repeated three times. GraphPad Prism 8 software (https://www.graphpad.com/) was used to calculate the standard deviation (SD), and a Student’s *t*-test analysis was performed.

### RNA extraction and qRT-PCR

A Plant RNA kit (OMEGA, Norcross, GA, USA) was used to extract sample RNAs, and a Reverse Transcription and cDNA Synthesis Kit (Clontech, Mountain View, CA, USA) was used to synthesize cDNA. Specific qRT-PCR primers for the internal reference gene *UBQ9* (Jin et al., 2019) and maize *NF-YA* subunit genes (Table S2) synthesized by Beijing Bomede Biotechnology Co., Ltd. were used to analyze the expression of *NF-YA* subunit genes with two hormone treated maize inbred line B73 plant samples collected at different times as templates. The qRT-PCR primers of the internal reference gene *UBQ1* (da Silva Santos et al., 2021) and *ZmPR*s (Table S3) were used for the expression analysis of *ZmPR*s with the leaf cDNAs of maize B73, *nfya01* and *nfya06* at the five-leaf stage as templates.

The reaction system was as follows: 7 μL of 2× M5 HiPer SYBR Premix EsTaq (with Tli RNaseH; TaKaRa, Dalian, China), 1 μL of cDNA template, 0.5 μL of forward primer, 0.5 μL of reverse primer and 5 μL of ddH_2_O. A fluorescence quantitative PCR instrument (CFX96 Real-time PCR Detection; BioRad, Hercules, CA, USA) was used for a total of 40 cycles, each of which was 95 °C for 30 s, 95 °C for 5 s and 60 °C for 30 s. Each qRT-PCR reaction was repeated three times, and the gene expression level was analyzed using the *Ct* value method (2^−ΔΔ*Ct*^). The SD of three replicates was calculated using GraphPad Prism 8 software, and the Student’s *t*-test analysis was performed.

## Results

### Bioinformatics analysis of maize *NF-YA* subunit genes

**Figure 1:**
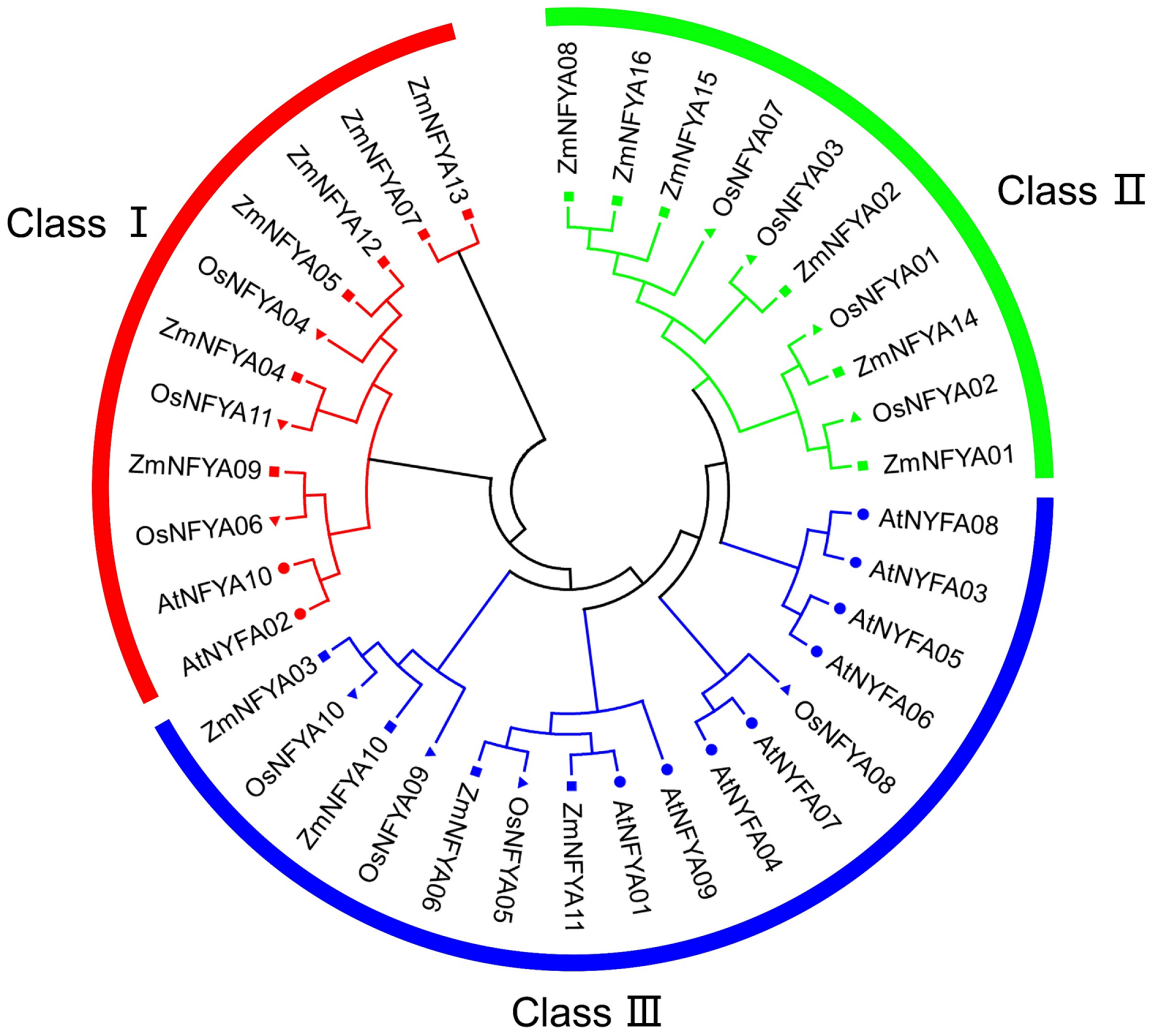
Phylogenetic relationships of *NF-YAs* in *Arabidopsis*, rice and maize.

In total, 16 maize *NF-YA* subunit genes were obtained from MaizeGDB and named *ZmNFYA01*–*16* in accordance with their chromosomal distribution. The 16 corresponding proteins differed in the number of amino acid (aa), relative molecular mass and isoelectric point. The lengths of the 16 NF-YA amino acid sequences were between 90 and 742 aa, with most being approximately 300 aa. The predicted isoelectric points indicated that most of these proteins were alkaline, with only the proteins encoded by *ZmNFYA15* and *ZmNFYA16* being acidic (Table 1). The 16 maize *NF-YA* subunit genes, 10 *Arabidopsis NF-YA* subunit genes and 11 rice *NF-YA* subunit genes could be divided into three groups: I, II and III. In maize, there were 6, 6 and 4 *NF-YA* subunit genes in I, II and III, respectively. Among them, *ZmNFYA01* was orthologous to *OsNFYA2*, *ZmNFYA06* was orthologous to *OsNFYA5*, *ZmNFYA09* was orthologous to *OsNFYA6*, *ZmNFYA10* was orthologous to *OsNFYA4*, and *ZmNFYA14* was orthologous to *OsNFYA1* (Fig. 1).

**Table 1:** Physicochemical properties of *NF-YA* subunit genes in maize.

Gene ID	Gene name	Chr	Start	End	AA	MW (Da)	pI
Zm00001d027874	*ZmNFYA01*	1	16,042,002	16,038,734	249	27,207.16	8.96
Zm00001d029489	*ZmNFYA02*	1	72,880,539	72,887,083	90	10,062.62	11.87
Zm00001d031063	*ZmNFYA03*	1	175,269,072	175,263,789	742	85,041.59	9.42
Zm00001d031092	*ZmNFYA04*	1	176,875,893	176,869,959	330	35,231.81	8.93
Zm00001d033215	*ZmNFYA05*	1	254,416,539	254,420,845	322	34,122.58	9.61
Zm00001d033602	*ZmNFYA06*	1	268,308,237	268,315,814	255	26,775.89	9.52
Zm00001d033773	*ZmNFYA07*	1	273,183,116	273,187,001	262	29,076.26	9.80
Zm00001d006835	*ZmNFYA08*	2	217,600,202	217,595,882	300	32,759.35	9.02
Zm00001d007882	*ZmNFYA09*	2	241,675,483	241,672,970	294	31,336.04	9.78
Zm00001d041491	*ZmNFYA10*	3	123,767,258	123,760,201	289	31,009.56	9.39
Zm00001d013501	*ZmNFYA11*	5	12,927,450	12,924,588	126	14,415.92	10.90
Zm00001d013676	*ZmNFYA12*	5	17,050,121	17,054,266	341	35,736.46	9.40
Zm00001d013856	*ZmNFYA13*	5	22,800,236	22,795,868	156	16,876.48	9.64
Zm00001d018255	*ZmNFYA14*	5	217,466,991	217,462,041	293	31,907.09	10.31
Zm00001d022109	*ZmNFYA15*	7	170,564,261	170,559,870	271	29,607.50	6.63
Zm00001d026305	*ZmNFYA16*	10	143,264,094	143,272,292	428	48,253.63	6.16

The maize *NF-YA* subunit genes were unevenly distributed among the chromosomes. Chromosome 1 contained the largest number (seven) of maize *NF-YA* subunit genes, *ZmNFYA01–7*. Chromosome 5 contained four maize *NF-YA* subunit genes: *ZmNFYA11–14*. Chromosome 2 contained two maize *NF-YA* subunit genes, *ZmNFYA08* and *ZmNFYA09*. Chromosomes 3, 7 and 10 contained only one maize *NF-YA* subunit gene each, whereas chromosomes 4, 6, 8 and 9 did not contain any maize *NF-YA* subunit genes (Fig. S3). The lengths of the *NF-YA* subunit gene sequences were quite different, with *ZmNFYA03*, *-07*, *-11* and *-13* having no 5′-UTR and 3′-UTR structures. Introns existed in the 5′-UTRs of 10 genes, and most genes had 4–6 exons, suggesting that they may share the same RNA splicing pattern (Fig. S4).

**Figure 2:**
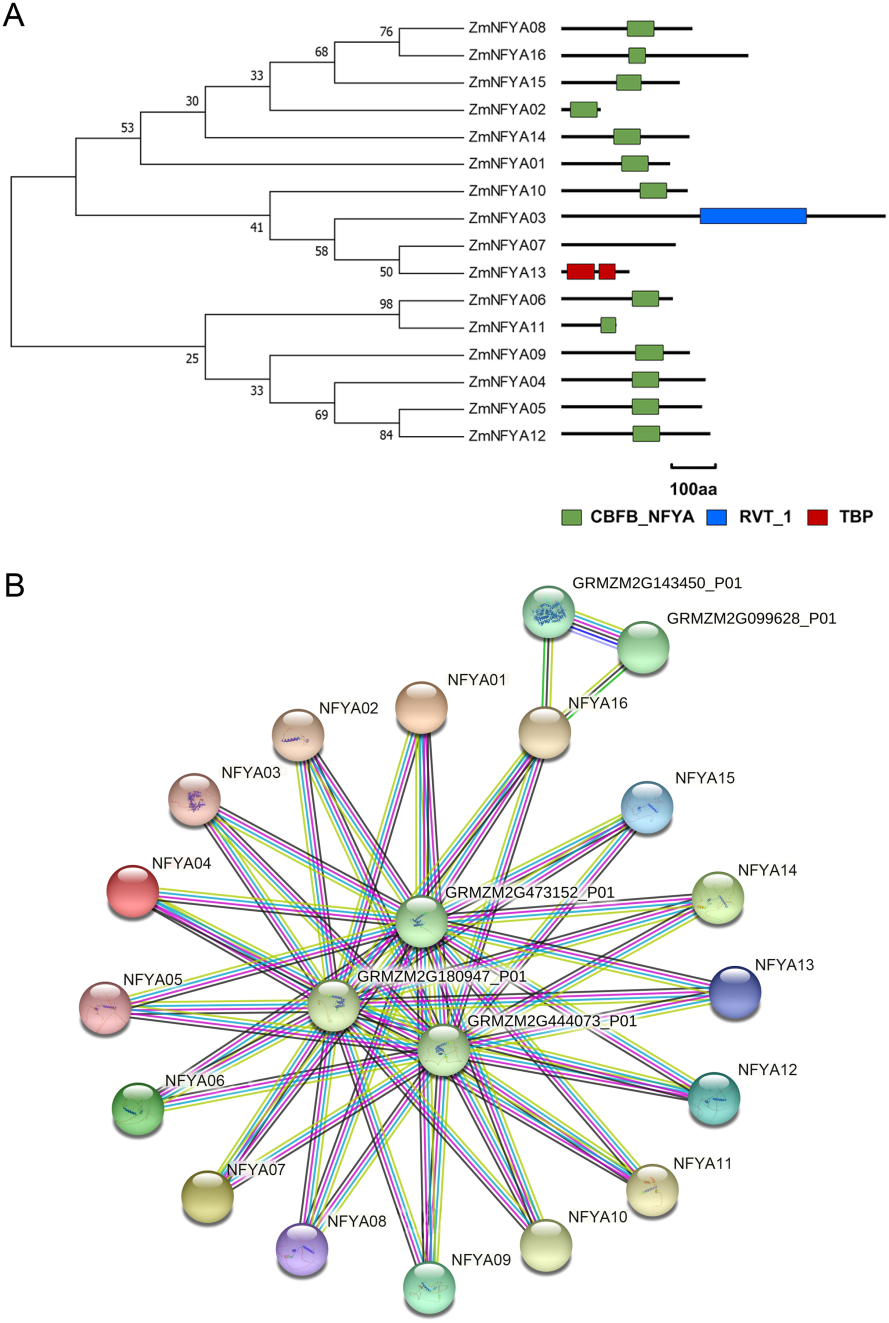
Conservative domain analysis and interaction protein network prediction of NF-YAs in maize. (A) Phylogenetic analysis and domain analysis of NF-YAs in maize. Green box: CBFB_NF-YA domain; blue box: reverse transcriptase domain; red box: TATA-binding protein domain. (B) The PPI network of ZmNFYAs detected by STRING. Empty nodes: proteins of unknown 3D structure; filled nodes: some 3D structure is known or predicted.

Among the 16 members in the NF-YA family, 13 contained a CBFB_NF-YA domain, ZmNFYA03 contained a reverse transcriptase domain, and ZmNFYA13 had a TATA-binding protein domain. ZmNFYA07 lacked any known domains (Fig. 2A). The CBFB_NF-YA domain plays a key role in binding the NF-YB subunit and specifically binds the CCAAT box.

ZmNFYA07 was predicted to be located in the chloroplast and cytoplasm, whereas ZmNFYA16 was predicted to be located in the mitochondrion and nucleus. The 14 remaining maize NF-YAs were predicted to be located in the nucleus (Table 2). Thus, most transcription factors were located in the nucleus, but they also played important roles in the mitochondrion and chloroplast.

**Table 2:** Subcellular localization prediction table for maize NF-YA subunit genes.

Gene name	Predicted location (s)
*ZmNFYA01*	Nucleus
*ZmNFYA02*	Nucleus
*ZmNFYA03*	Nucleus
*ZmNFYA04*	Nucleus
*ZmNFYA05*	Nucleus
*ZmNFYA06*	Nucleus
*ZmNFYA07*	Chloroplast, Cytoplasm
*ZmNFYA08*	Nucleus
*ZmNFYA09*	Nucleus
*ZmNFYA10*	Nucleus
*ZmNFYA11*	Nucleus
*ZmNFYA12*	Nucleus
*ZmNFYA13*	Nucleus
*ZmNFYA14*	Nucleus
*ZmNFYA15*	Nucleus
*ZmNFYA16*	Mitochondrion, Nucleus

All NF-YA proteins were predicted to interact with proteins encoded by GRMZM2G180947_P01, GRMZM2G473152_P01 and GRMZM2G444073_P01. In addition, GRMZM2G143450_P01 and GRMZM2G099628_P01 may interact with ZmNFYA16. These two proteins may encode a methionine-tRNA ligase, which indicates that ZmNFYA16 may play a role in translation (Fig. 2B).

### Existing data-based expression patterns of *NF-YA* subunit genes in maize

**Figure 3:**
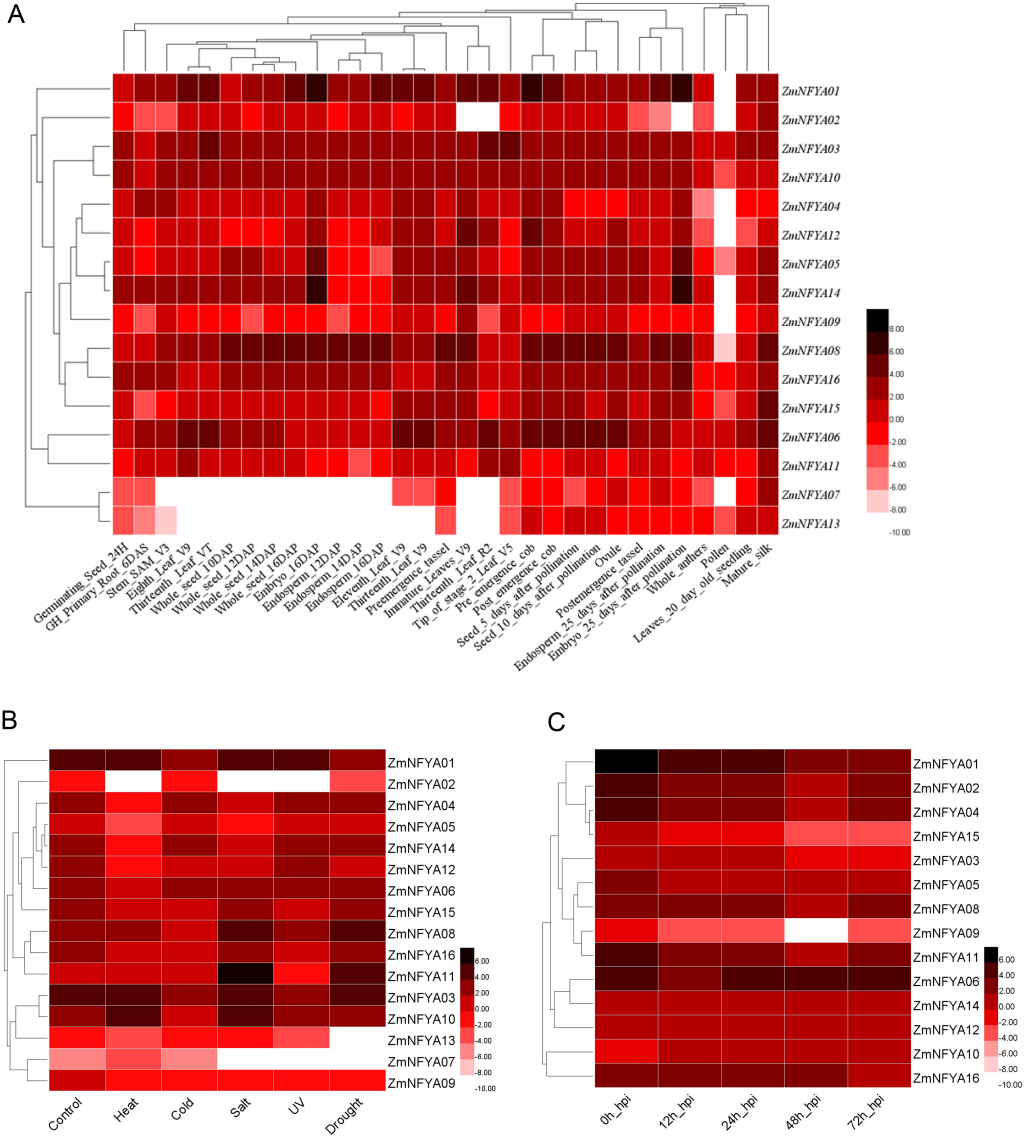
Existing data-based expression patterns of *NF-YA* subunit genes in maize. (A) Hierarchical clustering of expression levels of *ZmNFYAs* in 31 tissues. (B) Hierarchical clustering of expression levels of *ZmNFYAs* under abiotic stress. (C) Hierarchical clustering of expression levels of *ZmNFYAs* under biotic stress. Deep color indicates high expression; light color indicates low expression level.

The expression levels of the 16 maize *NF-YA* subunit genes in the same tissues at different developmental stages were significantly different (*P* < 0.05). The expression of the same gene varied in different tissues at different stages. *ZmNFYA01* was highly expressed at 25 d of embryonic development, and its expression levels in most tissues were significantly higher than those of the other genes, indicating that it played important roles in maize growth and development. *ZmNFYA14* was highly expressed at 16 d and 25 d of embryonic development. *ZmNFYA08* was highly expressed during embryonic development, endosperm development and seed germination. Low expression levels of *ZmNFYA02*, *ZmNFYA07* and *ZmNFYA13* were observed in all the tissues we studied (Fig. 3A).

The expression of *ZmNFYA08* was significantly up-regulated under salt and drought stresses. The expression of *ZmNFYA11* was similar under salt and drought treatments. *ZmNFYA10* was up-regulated under heat and salt stresses. *ZmNFYA02* was significantly down-regulated under heat, salt and UV stresses. The expression levels of *ZmNFYA05*, *ZmNFYA12* and *ZmNFYA14* decreased significantly under the heat stress. *ZmNFYA07* expression was low and maintained without significant change under the various stresses (Fig. 3B).

The expression levels of *ZmNFYA01* and *ZmNFYA15* were the highest at 0 h after the fungal infection, and then decreased gradually. The expression levels of *ZmNFYA02*, *-04–06*, *-08*, *-09*, *-11*, *-12* and *-14* all showed a trend of first decreasing and then increasing. The expression of *ZmNFYA03* and *ZmNFYA10* increased first, then decreased and finally increased. The expression of *ZmNFYA16* did not change significantly at 0–48 h after infection, but it decreased at 72 h after infection (Fig. 3C). Thus, the expression levels of *NF-YA* subunit genes in maize varied during *F. graminearum* infection, indicating that *NF-YA* subunit genes were involved and played important roles in maize disease resistance.

### Expression of maize *NF-YA* subunit genes under hormone treatment

**Figure 4:**
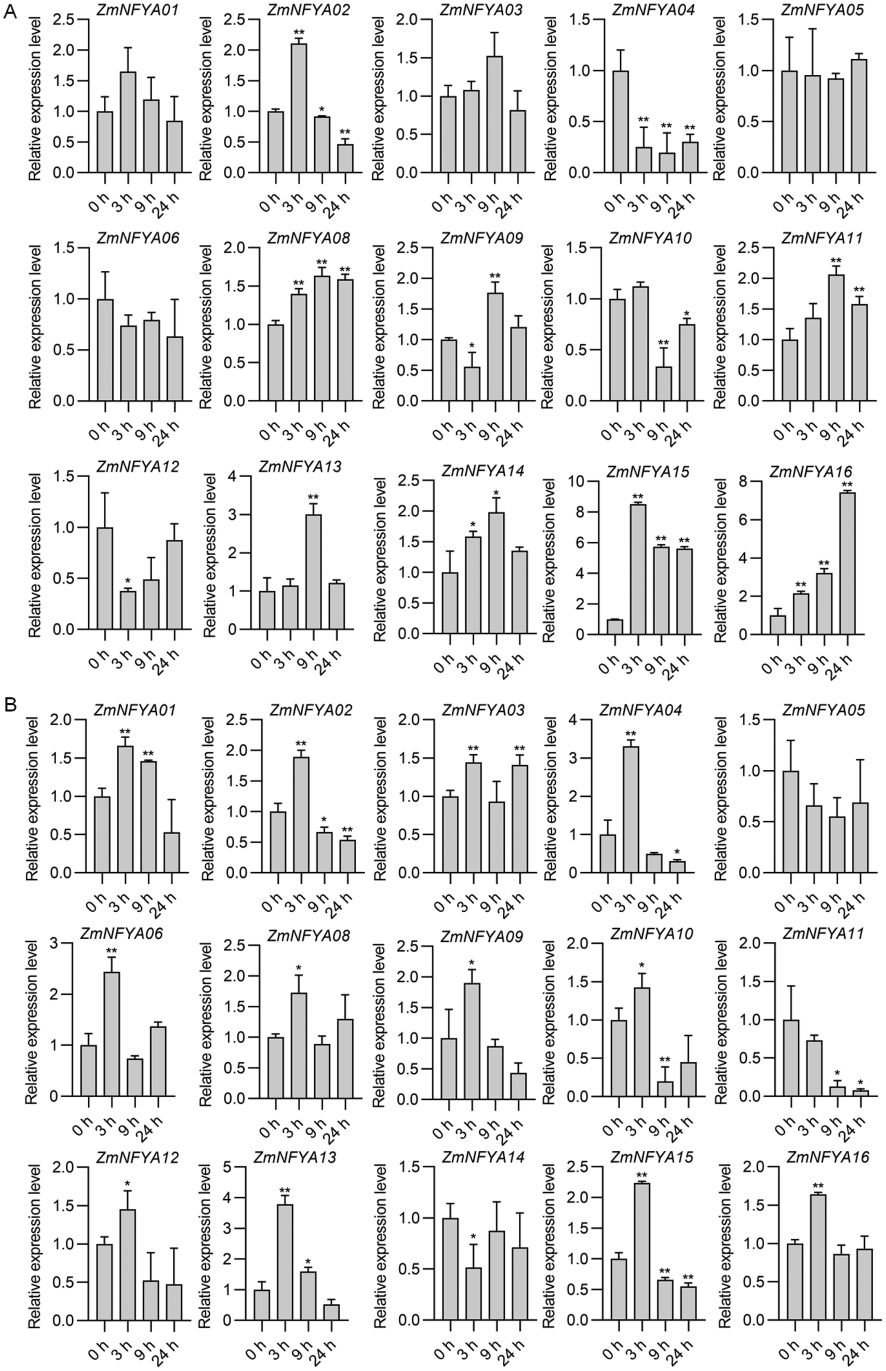
Expression changes in *ZmNFYAs* following hormone treatments. Expression changes after independent SA (A) and JA (B) treatments. Horizontal coordinates represent processing time. The experiments were repeated three times with similar results. Error bars indicate standard deviations. Asterisks indicate significant differences as assessed by Student’s *t*-tests (*P < 0.05; **P < 0.01).

With the SA treatment, maize *NF-YA* subunit genes were divided into four categories based on their expression. In the first type, the expression increased first and then decreased. The expression levels of *ZmNFYA01*, *-02* and *-15* peaked at 3 h after treatment, and then decreased gradually. The expression levels of *ZmNFYA03*, *-11*, *-13* and *-14* peaked at 9 h after treatment and then decreased. In the second type, the expression decreased first and then increased. The expression levels of *ZmNFYA04* and *ZmNFYA05* were lowest at 9 h after treatment and then increased slightly. The expression of *ZmNFYA12* was lowest at 3 h after treatment and then increased to the initial level. In the third type, the expression of the gene such as *ZmNFYA08* and *ZmNFYA16* continued to increase was maintained at a high level after the SA treatment. In the fourth type, the expression fluctuated after treatment. For example, the expression levels of *ZmNFYA06* and *ZmNFYA09* decreased first, then increased and finally decreased. The expression of *ZmNFYA10* increased first and then decreased to its lowest level before increasing again (Fig. 4A).

Under the JA treatment, the maize *NF-YA* subunit genes were divided into three categories based on their expression patterns. In the first type, after the JA treatment, the gene expression peaked at 3 h after treatment and then gradually decreased, which included *ZmNFYA01*, *-02*, *-04*, *-09*, *-12*, *-13*, *-15* and *-16*. In the second type, the expression increased first and then decreased before increasing again. The expression of *ZmNFYA03*, *-06*, *-08* and *-10* peaked at 3 h after treatment, decreased to their lowest levels at 9 h and then increased. In the third type, which included *ZmNFYA05* and *ZmNFYA11*, expression decreased after the JA treatment. In addition, the expression of *ZmNFYA14* decreased first and then increased before decreasing again (Fig. 4B). Thus, the *NF-YA* subunit genes may be involved and play important roles in SA and JA signaling pathways.

### Positive regulation of maize disease resistance by *ZmNFYA01* and *ZmNFYA06*

**Figure 5:**
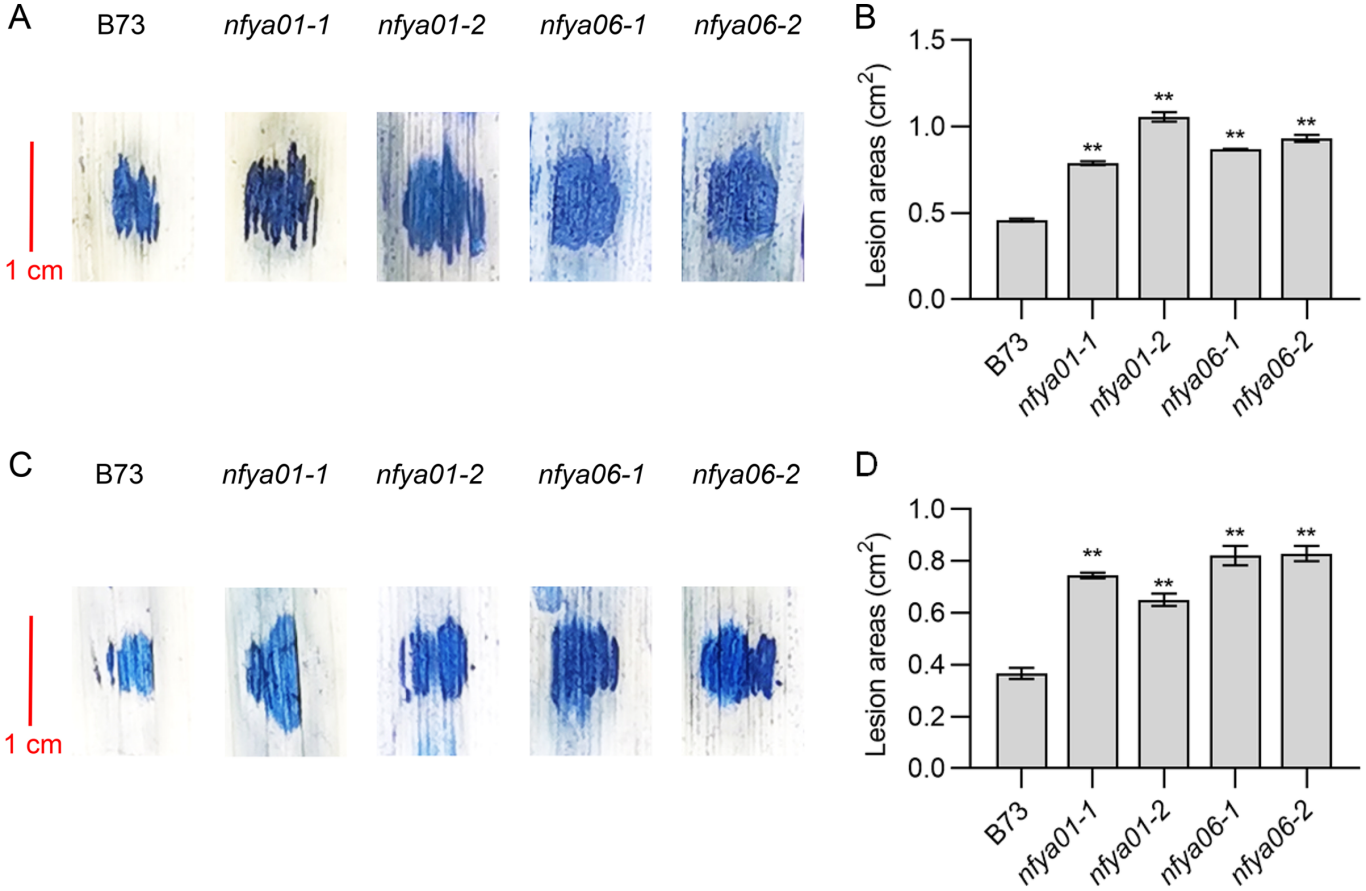
Resistance analyses of *nfya01* and *nfya06*. The leaves stained with trypan blue of B73, *nfya01* and *nfya06* inoculation with (A) *S. turcica* and (C) *C. heterostrophus*. (B and D) Measurement of the lesion areas on leaves shown in A and C, and the experiments were repeated three times with similar results. Error bars indicate standard deviations. Asterisks indicate significant differences as assessed by Student’s *t*-tests (*P < 0.05; **P < 0.01).

The lesion areas of *nfya01* and *nfya06* plants inoculated with *S. turcica* and *C. heterostrophus* were significantly larger than those of inbred line B73 (Figs. 5A–5D). Compared with B73, the sensitivity of *nfya01* and *nfya06* to *S. turcica* and *C. heterostrophus* was enhanced, indicating that *ZmNFYA01* and *ZmNFYA06* positively regulate the disease resistance of maize and provide broad-spectrum resistance to pathogenic fungi, thereby playing important roles in maize resistance to pathogen infection.

### Expression analysis of *ZmPRs* in *nfya01* and *nfya06*

**Figure 6:**
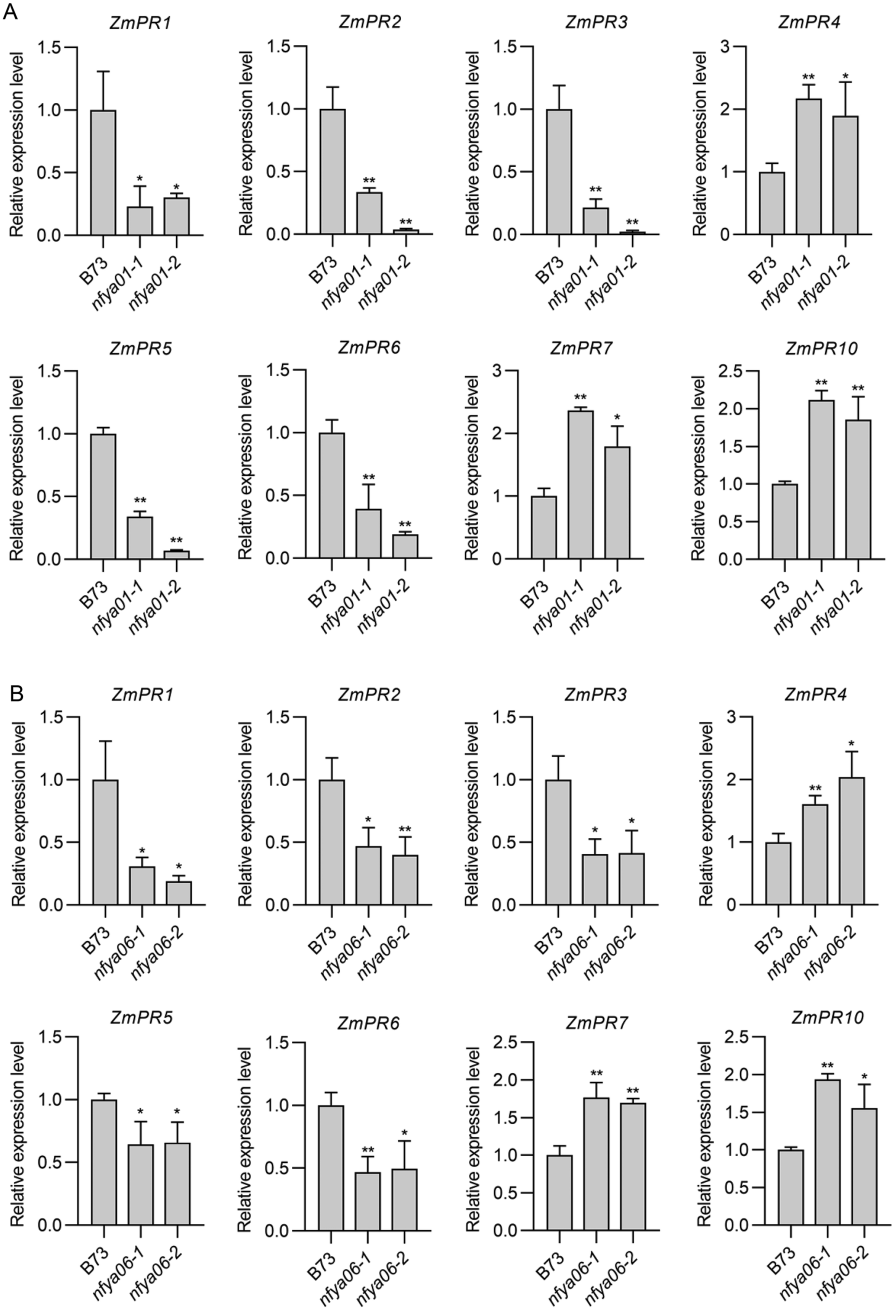
Expression analyses of *ZmPRs* in *nfya01* and *nfya06*. Expression levels of *ZmPR*s in (A) *nfya01* and (B) *nfya06*. The experiments were repeated three times with similar results. Error bars indicate standard deviations. Asterisks indicate significant differences as assessed by Student’s *t*-test (*P < 0.05; **P < 0.01).

Compared with in B73, the expression of *ZmPR1–3*, *-5* and *-6* in *nfya01* and *nfya06* plants were significantly down-regulated (*P* < 0.05), whereas the expression of *ZmPR4*, *-7* and *-10* were significantly up-regulated (*P* < 0.05) (Figs. 6A and 6B), indicating that *ZmNFYA01* and *ZmNFYA06* affected the expression of *ZmPRs* and suggesting that *ZmNFYA01* and *ZmNFYA06* participated in plant disease resistance by regulating the expression of *ZmPRs*. The expression patterns of different *ZmPRs* in *nfya01* and *nfya06* plants were consistent, indicating that *ZmNFYA01* and *ZmNFYA06* had similar functions.

## Discussion

The roles of *NF-YA* subunit genes in the molecular mechanisms involved in maize responses to pathogen infection have been rarely reported. In this study, 16 *NF-YA* subunit genes were obtained by phylogenetic analysis and divided into three categories, with two more *NF-YA* members identified than what Zhang et al. (2016) have done. The eukaryotic 5′-UTR is critical for ribosome recruitment to the messenger RNA (mRNA) and in start codon choice. Additionally, it plays major roles in the control of translation efficiency and shaping the cellular proteome (Hinnebusch, Ivanov & Sonenberg, 2016). The 3′-UTRs of mRNAs regulate mRNA-based processes, such as mRNA localization, mRNA stability and translation. In addition, 3′-UTRs can establish 3′-UTR-mediated PPIs, and thus transmit genetic information encoded in 3′-UTRs to proteins. This function regulates diverse protein features, including protein complex formation or posttranslational modifications, but it may also alter protein conformations. Therefore, 3′-UTR-mediated information transfer can regulate protein features that are not encoded in the amino acid sequence (Mayr, 2019). However, *ZmNFYA03*, *-07*, *-11* and *-13* do not have 5′-UTR and 3′-UTR structures. Thus, further research is required to determine how they perform these functions.

The predicted PPI network indicated that all 16 NF-YA proteins interact with GRMZM2G180947_P01, GRMZM2G473152_P01 and GRMZM2G444073_P01 (Fig. 2B), which were named ZmNF-YB4 (GRMZM2G180947_P01), ZmNF-YB6 (GRMZM2G473152_P01) and ZmNF-YB9 (GRMZM2G444073_P01), respectively (Zhang et al., 2016). The naming is consistent with the binding of CBFB_NF-YA, a conserved domain in the associated *NF-YA* subunit genes, to the NF-YB subunit. Yang et al. (2022) confirmed that ZmNF-YB16 interacts with ZmNF-YC17 through its histone-folding domain to form a heterodimer in the cytoplasm. Then, the complex enters the nucleus under osmotic-stress conditions to form a heterotrimer with ZmNF-YA1 or ZmNF-YA7 (ZmNFYA08 in this study), forming the first identified ZmNF-Y transcriptional regulatory complex in maize (Yang et al., 2022). These results laid a foundation for elucidating the functions and regulatory mechanisms of maize *NF-YA* subunit genes. However, the specific functions of *NF-YA* subunit genes and their relationships with NF-YBs and NF-YCs still require further study.

The overexpression of *ZmNF-YA1* enhances drought and salt tolerance and promotes root development in maize, whereas the *zmnf-ya1* mutant shows drought and salt sensitivity (Yang et al., 2022). In this study, *ZmNFYA01* was highly expressed during embryogenesis and positively regulated maize disease resistance, suggesting that it played important roles in maize growth and development. However, its regulatory mechanisms need further study.

## Conclusions

In summary, 16 maize *NF-YA* subfamily genes were identified, and their expression levels in the same tissue at different developmental stages revealed a pattern, and the gene expression levels changed significantly under biotic and abiotic stress, including SA and JA treatments. *ZmNFYA01* and *ZmNFYA06* positively regulated maize resistance, and provided broad-spectrum resistance to pathogenic fungi. Compared with in B73, the expression levels of the *ZmPRs* in *nfya01* and *nfya06* plants were changed significantly, suggesting that this is part of the regulation of maize disease resistance. The important roles of *NF-YA* subunit genes in maize growth, development and resistance to biotic and abiotic stresses have been preliminarily determined.

## Supplemental Information

10.7717/peerj.14306/supp-1 Supplemental Information 1 PCR identification and qRT-PCR identification of *nfya01*. (A) PCR identification, 1: nfya01F+nfya01R, 2: nfya01F+Mu67, 3: nfya01R+Mu67. (B) qRT-PCR identification. The experiments were repeated three times with similar results. Error bars indicate standard deviations. Asterisks indicate significant differences as assessed by Student’s *t*-tests (*P < 0.05; **P < 0.01).

10.7717/peerj.14306/supp-2 Supplemental Information 2 PCR identification and qRT-PCR identification of *nfya06*. (A) PCR identification, 1: nfya06F+nfya06R, 2: nfya06F+Mu67, 3: nfya06R+Mu67. (B) qRT-PCR identification. The experiments were repeated three times with similar results. Error bars indicate standard deviations. Asterisks indicate significant differences as assessed by Student’s *t*-tests (*P < 0.05; **P < 0.01).

10.7717/peerj.14306/supp-3 Supplemental Information 3 Chromosomal localization of *NF-YAs* in maize. Different genes are represented by dots of different colors. Stripes represent gene density.

10.7717/peerj.14306/supp-4 Supplemental Information 4 Exon-intron organization of *NF-YAs* in maize. Blue boxes: UTRs; green boxes: Exons; black lines: introns.

10.7717/peerj.14306/supp-5 Supplemental Information 5 Primers for PCR identification of *nfya01* and *nfya06*.

10.7717/peerj.14306/supp-6 Supplemental Information 6 The qRT-PCR primers for maize *NF-YA* subunit genes.

10.7717/peerj.14306/supp-7 Supplemental Information 7 The qRT-PCR primers for maize *ZmPRs*.

10.7717/peerj.14306/supp-8 Supplemental Information 8 Raw data for gene expression.

10.7717/peerj.14306/supp-9 Supplemental Information 9 Raw data for PRs expression.

## Additional Information and Declarations

### Data Availability

The following information was supplied regarding data availability: The raw data is available in the Supplemental Files.
