# Supplementary figures and images for: Identification and expression analysis of maize NF-YA subunit genes
Source: PeerJ. 2022 Nov 7;10:e14306. doi: 10.7717/peerj.14306 (PMC9648346; doi:10.7717/peerj.14306)

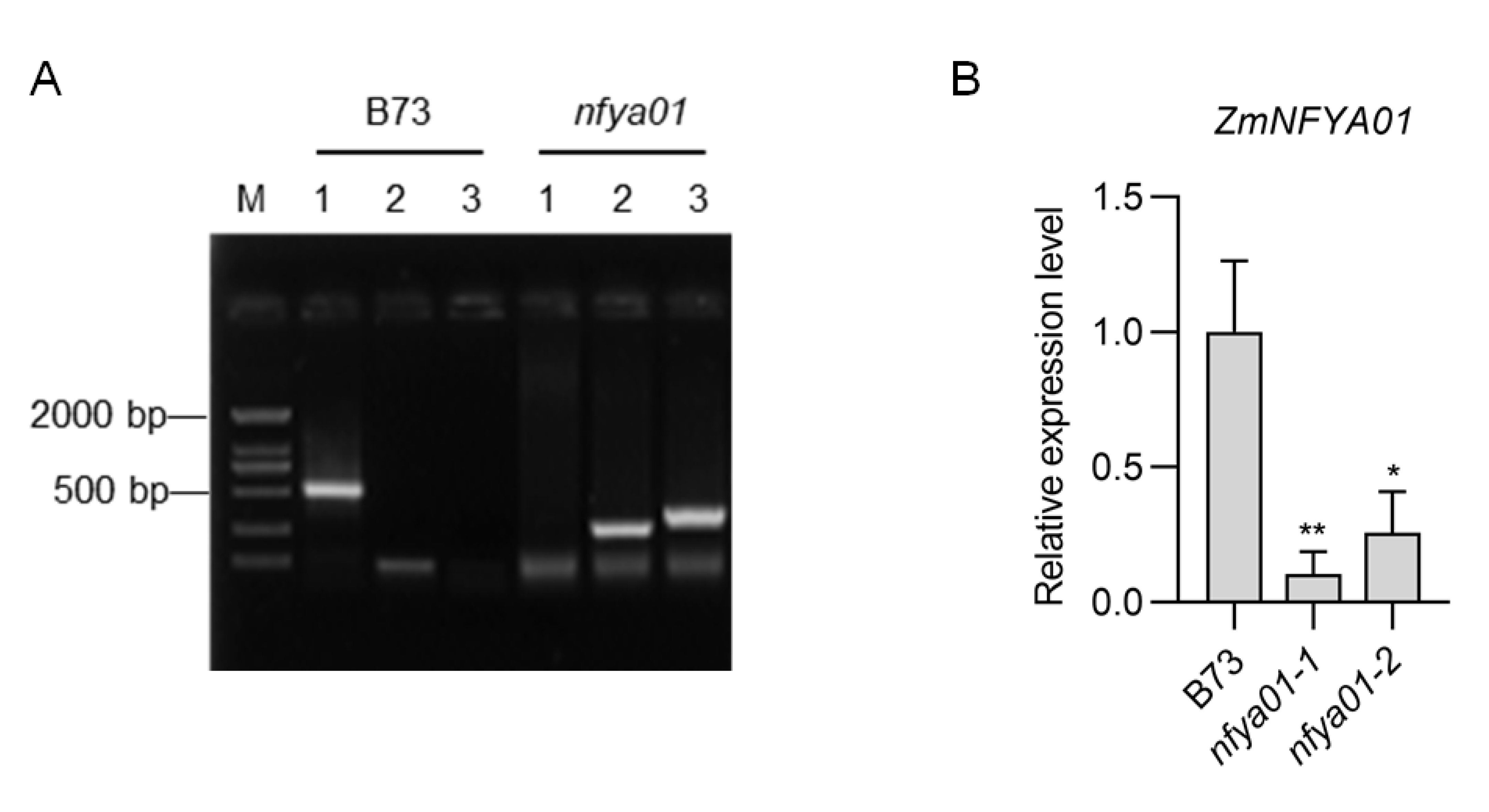

Supplement: Supplemental Information 1 — (A) PCR identification, 1: nfya01F+nfya01R, 2: nfya01F+Mu67, 3: nfya01R+Mu67. (B) qRT-PCR identification. The experiments were repeated three times with similar results. Error bars indicate standard deviations. Asterisks indicate significant differences as assessed by Student’s t-tests (*P < 0.05; **P < 0.01). [file peerj-10-14306-s001.jpg]

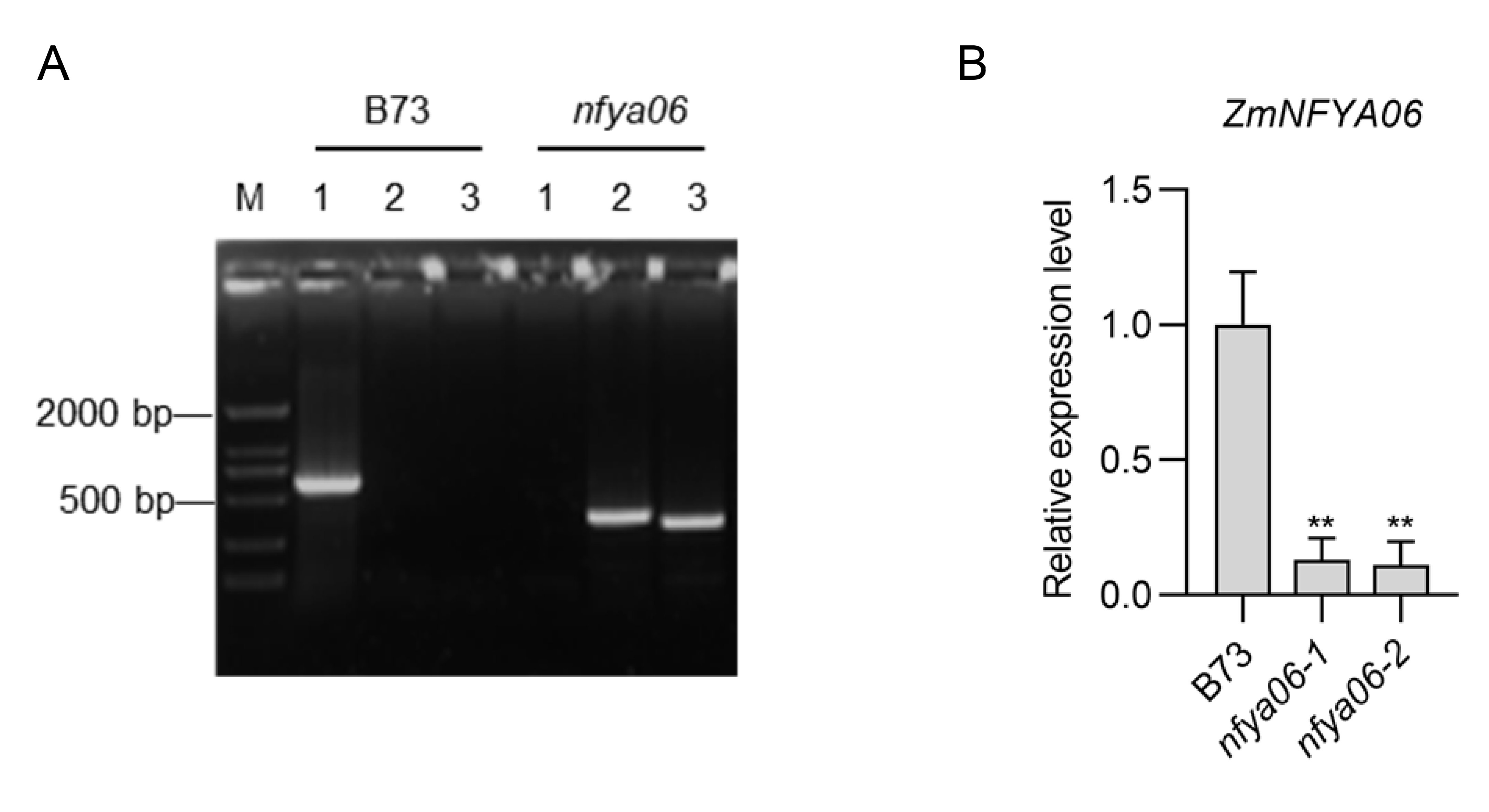

Supplement: Supplemental Information 2 — (A) PCR identification, 1: nfya06F+nfya06R, 2: nfya06F+Mu67, 3: nfya06R+Mu67. (B) qRT-PCR identification. The experiments were repeated three times with similar results. Error bars indicate standard deviations. Asterisks indicate significant differences as assessed by Student’s t-tests (*P < 0.05; **P < 0.01). [file peerj-10-14306-s002.jpg]

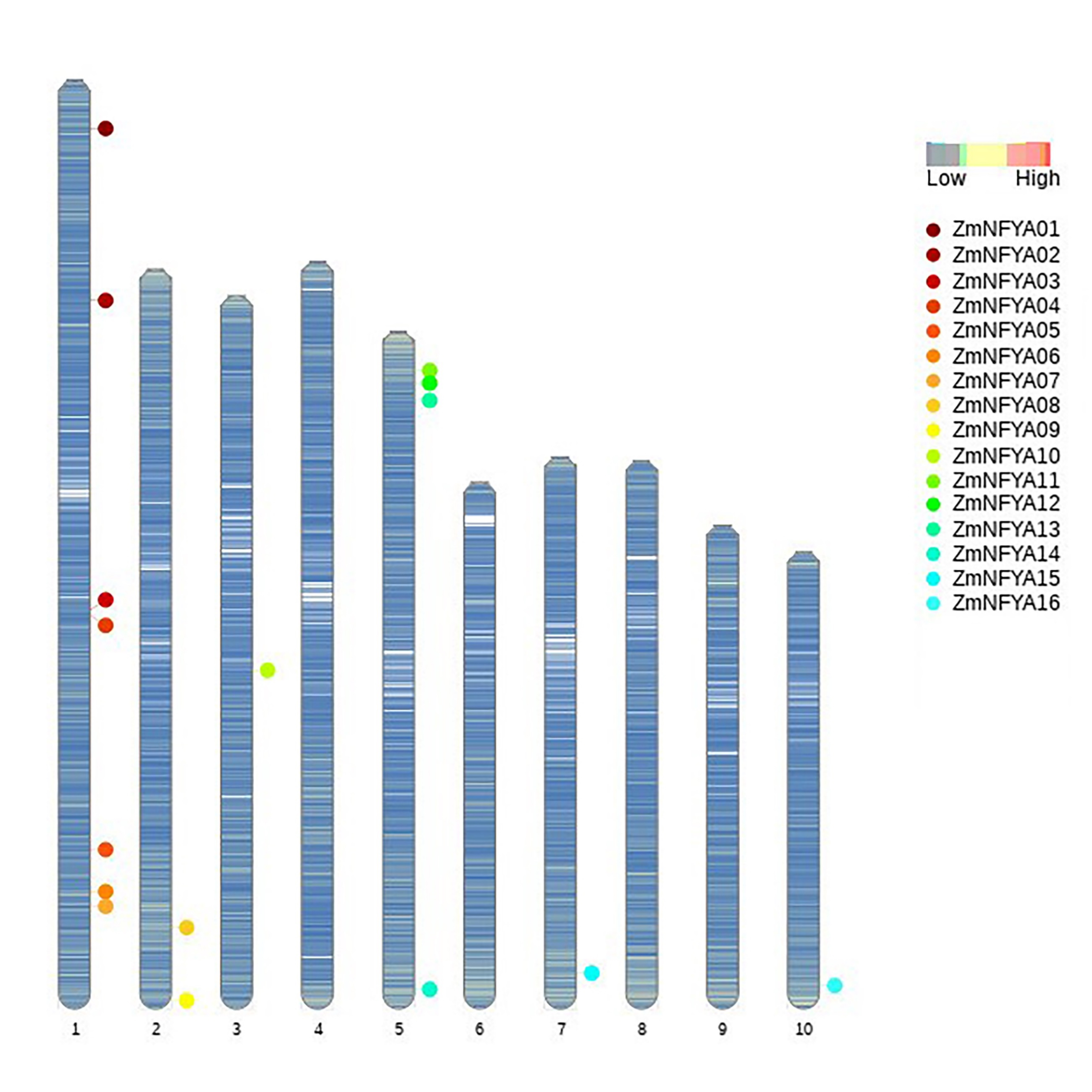

Supplement: Supplemental Information 3 — Different genes are represented by dots of different colors. Stripes represent gene density. [file peerj-10-14306-s003.jpg]

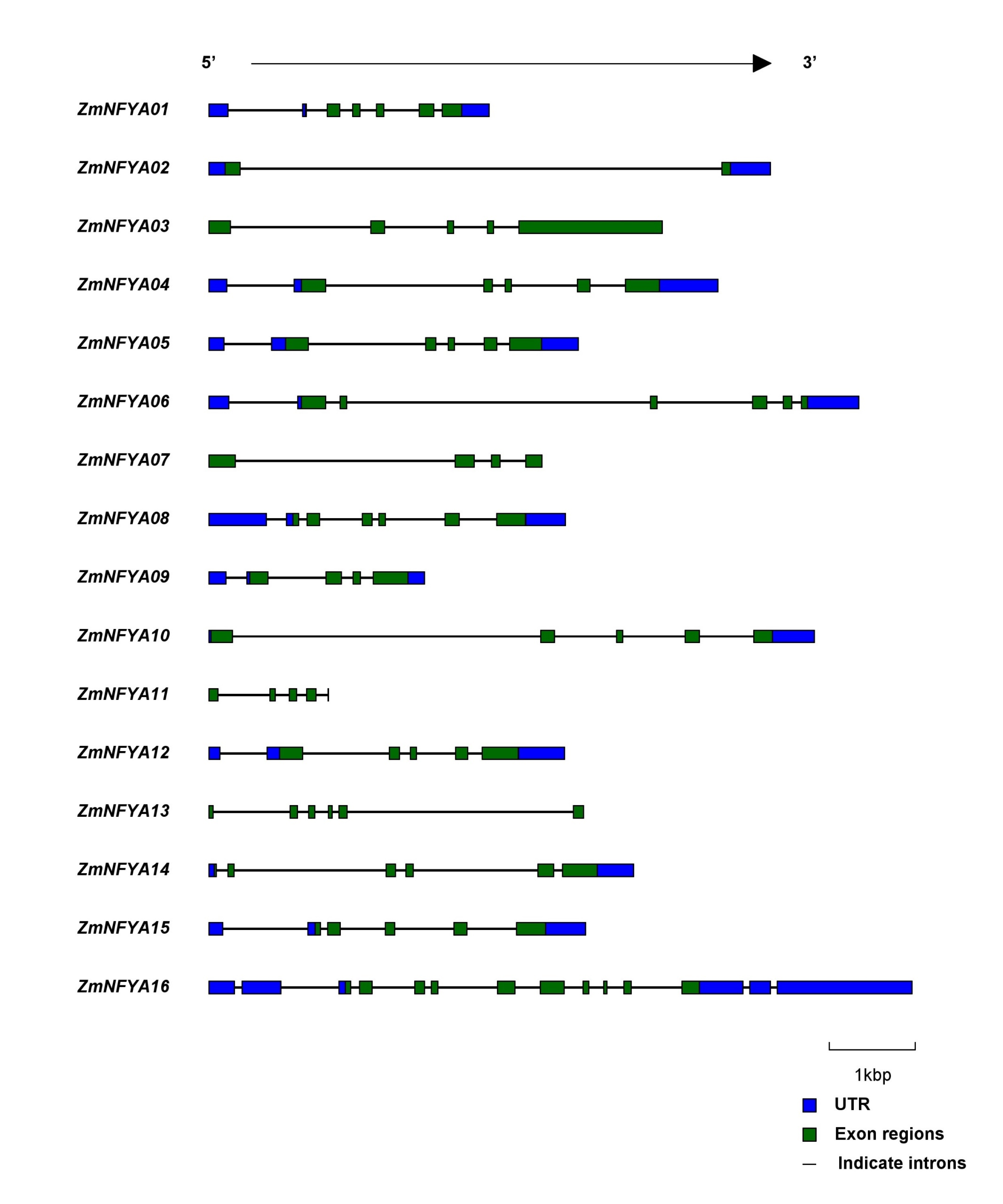

Supplement: Supplemental Information 4 — Blue boxes: UTRs; green boxes: Exons; black lines: introns. [file peerj-10-14306-s004.jpg]
