# Supplementary material for: Identification and expression analysis of maize NF-YA subunit genes
Source: PeerJ. 2022 Nov 7;10:e14306. doi: 10.7717/peerj.14306 (PMC9648346; doi:10.7717/peerj.14306)
Supplement: Supplemental Information 5 [file peerj-10-14306-s005.docx]

**Table S1** Primers for PCR identification of *Zmnfya01* and *Zmnfya06*

| Primer name | Sequence (5′-3′) |
| --- | --- |
| Mu67 | GAAGCCAACGCCAWCGCCTCYATTTCGTCGAAT |
| NFYA01MuF | GGAGCAGCTCTTTTACCTCTTTTAG |
| NFYA01MuR | CACAAATCGTCCAGAAGAACCGAGA |
| NFYA06MuF | CCGAGACACTTACAATAATCCTTGC |
| NFYA06MuR | ATTCTCCCTCCACTCGACCAACAAA |
