# Supplementary material for: Identification and expression analysis of maize NF-YA subunit genes
Source: PeerJ. 2022 Nov 7;10:e14306. doi: 10.7717/peerj.14306 (PMC9648346; doi:10.7717/peerj.14306)
Supplement: Supplemental Information 6 [file peerj-10-14306-s006.docx]

**Table S2** The qRT-PCR primers for maize NF-YA subunit genes

| Gene name | Primer name | Sequence (5′–3′) |
| --- | --- | --- |
| *UBQ9* | UBQ9-qF | TACAGTTCTACAAGGTGGACGAC |
|  | UBQ9-qR | GCAGTAGTGGCGGTCGAAGT |
| *ZmNFYA01* | NFYA1-qF | AACCAGTGAGACGAGCCATC |
|  | NFYA1-qR | AGGCTTCGGATTATGTGCGT |
| *ZmNFYA02* | NFYA2-qF | ACACTAGGGTACCATTGCCAG |
|  | NFYA2-qR | GCTTCCGACCTTTCACCAGT |
| *ZmNFYA03* | NFYA3-qF | GTTGAAGTGGCGACAAGCTG |
|  | NFYA3-qR | TATCCAGCGCAACATACGCA |
| *ZmNFYA04* | NFYA4-qF | TCCTCAACCTCTCCGTGGAT |
|  | NFYA4-qR | CTGATGGCGTGACTCATGGA |
| *ZmNFYA05* | NFYA5-qF | ATGGCCGTTTCGAGATTGGT |
|  | NFYA5-qR | CAGCTGTCGCATTTAGTGGC |
| *ZmNFYA06* | NFYA6-qF | ATGAGCAGCATGGAGTCGC |
|  | NFYA6-qR | GTCACTCGCGGCTCCTTTAG |
| *ZmNFYA08* | NFYA8-qF | ACCAGCAGTATCAGGCATCG |
|  | NFYA8-qR | TTCAAGCCTCCTCGACCTTG |
| *ZmNFYA09* | NFYA9-qF | GGTGCTTAACAACGACAGCG |
|  | NFYA9-qR | AACTGCTTGGCGTTCACGTA |
| *ZmNFYA10* | NFYA10-qF | TGGCTTACCTTTGCCTACCG |
|  | NFYA10-qR | GCGCAACTCCGTTCTGAATC |
| *ZmNFYA11* | NFYA11-qF | ACATCGCAGACACCTGCTTT |
|  | NFYA11-qR | GCTCCTCGGACACACATCTT |
| *ZmNFYA12* | NFYA12-qF | TCGCGGAATACAATGGTCGT |
|  | NFYA12-qR | GCTTTCTGCCTTTGGTGAGC |
| *ZmNFYA13* | NFYA13-qF | AACAGTGCTCGTAACGCAGA |
|  | NFYA13-qR | GAAACCGAGCTTCTGGACGA |
| *ZmNFYA14* | NFYA14-qF | TTACGTGCCAAGCTAGAGGC |
|  | NFYA14-qR | CTGCTGGAGCTGCTTAGTGT |
| *ZmNFYA15* | NFYA15-qF | AGTCCGGTTATGTTGCCTGC |
|  | NFYA15-qR | AGTTTAGCACGTGTCTGCCT |
| *ZmNFYA16* | NFYA16-qF | TGCTGATTTGGCTGCAAAGG |
|  | NFYA16-qR | TAGTGAGCACTCATTGCGCT |
