# Supplementary material for: Identification and expression analysis of maize NF-YA subunit genes
Source: PeerJ. 2022 Nov 7;10:e14306. doi: 10.7717/peerj.14306 (PMC9648346; doi:10.7717/peerj.14306)
Supplement: Supplemental Information 7 [file peerj-10-14306-s007.docx]

**Table S3** The qRT-PCR primers for maize *ZmPRs*

| Gene name | Primer name | Sequence (5′–3′) |
| --- | --- | --- |
| *UBQ1* | UBQ1-qF | CCGACCAGCAGCGCC |
|  | UBQ1-qR | GTAGTCCGCGAGAGTGCG |
| *ZmPR1* | ZmPR1-qF | CAGAACGCGGCGCGCGCGTC |
|  | ZmPR1-qR | TGCCGCTGCCCCAGAAGA |
| *ZmPR2* | ZmPR2-qF | ACGCTGAAGGTGACTCCG |
|  | ZmPR2-qR | GTCGTGGAACGTCTTGGTG |
| *ZmPR3* | ZmPR3-qF | TTTCCTCCTGCTGGATTGTCA |
|  | ZmPR3-qR | TAGCCCTGCCCGTTGGTGTC |
| *ZmPR4* | ZmPR4-qF | TGCCTCCAGGTGCGAATG |
|  | ZmPR4-qR | TGCCGACTACGTTGTGAGTGC |
| *ZmPR5* | ZmPR5-qF | TCGGTCATCGACGGCTACAA |
|  | ZmPR5-qR | CGGGCAGAAGGTGACTTGGTAG |
| *ZmPR6* | ZmPR6-qF | ACTGTTGAGATCGCCTCG |
|  | ZmPR6-qR | GAAGTTGAACTGCCTGACG |
| *ZmPR7* | ZmPR7-qF | CTTGCAGTCATGCCGTTCA |
|  | ZmPR7-qR | CGGGCTCCACCTTGATGT |
| *ZmPR10* | ZmPR10-qF | CACCTCAGTCATGCCGTTCA |
|  | ZmPR10-qR | CCCTCGATGAGCGTGTTCTT |
